# Prevalence of and factors related to the use of antidepressants and benzodiazepines: results from the Singapore Mental Health Study

**DOI:** 10.1186/1471-244X-13-231

**Published:** 2013-09-23

**Authors:** Mythily Subramaniam, Vincent YF He, Janhavi A Vaingankar, Edimansyah Abdin, Siow Ann Chong

**Affiliations:** 1 Research Division, Institute of Mental Health, Buangkok Green Medical Park, 10 Buangkok View, Singapore 539747, Singapore; 2 Research Division, Institute of Mental Health, Singapore, Singapore

**Keywords:** Antidepressant, Benzodiazepine, Major depressive disorder, Alcohol use disorder, Survey

## Abstract

**Background:**

Prescription and use of antidepressants and benzodiazepines are common in the general population. Prescription of psychotropic drugs is a complex process: patient, physician and healthcare characteristics mediate, interact and influence it. The current study aimed to establish the prevalence and factors associated with the use of antidepressants (ADs) and benzodiazepines (BZDs) in Singapore.

**Methods:**

The Singapore Mental Health Study (SMHS) was a nationally representative survey of Singapore Residents aged 18 years and above. Face-to-face interviews were conducted from December 2009 to December 2010. The diagnoses of mental disorders were established using the Composite International Diagnostic Interview version 3.0 (CIDI-3.0). The pharmacoepidemiology section was used to collect information on medication use.

**Results:**

The overall prevalence estimates for ADs and BZDs use during the 12 months prior to the interview were 1.1% and 1.2% respectively. In all, 2.0% had used ADs and/or BZDs. ‘Help seeking for emotional or mental health problems’ was the most important predictor for the use of ADs and BZDs—help seekers were much more likely to use ADs (adjusted OR: 31.62, 95% CI: 13.36–74.83) and more likely to use BZDs than non-help seekers in the previous 12 months (adjusted OR: 34.38, 95% CI: 12.97–91.16). Only 27.6% of those with 12-month major depressive disorder (MDD) had sought formal medical help for their problems and ADs were being used by just over a quarter of this ‘help-seeking group’ (26.3%).

**Conclusions:**

We found that the use of ADs and BZDs in our population was relatively low, and ‘help-seeking’ was the most important predictor of the use of ADs and BZDs. In concordance with research from other Western countries, use of ADs was low among those with 12-month MDD.

## Background

Population based surveys from Europe and North America have suggested that prescription rates of psychotropic drugs range from 10 to 15% in the population [1-3], with the most common being the prescription and use of antidepressants (ADs) and benzodiazepines (BZDs). A study of six countries across Europe found that 4.38% and 9.17% of the total sample reported the use of ADs and BZDs in the past 12-months [4].

Prescription of psychotropic drugs is a complex process: patient, physician and healthcare characteristics mediate, interact and influence it. It has been consistently reported that the consumption of psychotropic medications increases with age [5-8], is higher among women [5,7,9], among those with lower education [4,5,8] and the unemployed [10,11]. Mojtabai [12] showed that patients seeking help for depression and anxiety disorders from psychiatrists received ADs more frequently than if they were seeing primary care physicians. The nature and setting of the healthcare service such as primary versus tertiary healthcare setting, public versus private institutions, medical insurance systems, also influence prescription patterns. These factors may interact and further influence the prescription and use of these medications for example, Bellantuono et al. [13] found the perception of a social problem by a General Practitioner (GP) doubled the probability of a psychotropic drug being prescribed in women but not men; Taggart et al. [14] found that male physicians prescribed psychotropic drugs at a significantly higher rate for female patients as compared to female physicians.

The growing phenomenon of the pervasive use of ADs has given rise to questions regarding the appropriateness of their prescription as well as the rising costs. On the other hand, epidemiological surveys have uncovered large treatment gaps among those with major depressive disorder (MDD). Results from the European Study of the Epidemiology of Mental Disorders (ESEMeD) [15] showed that 63.5% of those with 12-month diagnosis of any mood disorder did not consult any formal health services, and among those who sought help, only 71% were prescribed a medication. The Singapore Mental Health Study (SMHS) showed that the treatment gap for those with lifetime MDD was 59.6% while that of dysthymia was 46.8% [16]. However, little is known in Singapore about the prevalence of AD use in the community. Previous studies on the prescription of psychotropic medications in Singapore were largely limited to populations undergoing psychiatric treatment in specialized clinical settings [17,18].

Singapore is a city-state in South-East Asia with a multi-ethnic population that totals 5 million. A principal feature of Singapore’s healthcare philosophy is the emphasis on individual responsibility and the need for co-payment for services provided. It has a dual healthcare system with a public and private sector where affordability of healthcare is ensured with the 3 Ms: Medisave, a national mandatory healthcare saving plan; Medishield, a national low cost medical insurance; and Medifund, where subsidies are provided for the needy through a national fund. Patients seeking treatment in public hospitals may apply for a range of subsidies on their total bill; the extent of subsidy received is subjected to guidelines set by the government to allocate limited resources to those who need them most. Mental health services are provided mainly by psychiatrists and primary care doctors. GPs provide 80% of the primary healthcare services, and doctors in government polyclinics provide the remaining 20%. Public hospitals (referred to as restructured hospitals) provide about 80% of the tertiary care in Singapore, while the remaining are provided by private hospitals.

The main objectives of this study were to establish the prevalence of AD and BZD use and their associated factors in Singapore. We examined the effect of socio-demographic factors, diagnosis of mood, anxiety or alcohol use disorders, disability due to mental disorders, help-seeking as well as the presence of comorbid chronic physical condition on the use of ADs and BZDs across a national sample in Singapore.

## Methods

### Sample

The Singapore Mental Health Study (SMHS) was a nationally representative survey of Singapore Residents aged 18 years and above. A disproportionate stratified sampling was used where the 3 main ethnic groups (Chinese, Malays, and Indians) were sampled in equivalent proportion of about 30% each and the remaining 10% belonged to ‘Other’ ethnic groups. This was to address the possibility of not getting an adequate sample in minority ethnic groups to accurately establish the prevalence of an uncommon disorder. The sample size was derived from a statistical power calculation for binary proportions using previously established prevalence rates of mental disorders in Singapore. We found the margin of error using this sample distribution for the overall prevalence estimate was between 1.5%—3.0%, while the margin of error for the strata defined by age and ethnic groups was 1.0—3.5%. As the margin of error (or precision) of a binary proportion depends on the estimate, we also computed the relative standard error (RSE), which was below 30%.

The respondents were randomly selected from a National registry and approached at the household address provided by the registry. The study was approved by the relevant institutional ethics committee (National Healthcare Group Domain Specific Review Board) and written informed consent was obtained from all respondents. Face-to-face interviews were conducted by trained lay interviewers, and the household survey was conducted from December 2009 to December 2010. 6,616 respondents completed the interview, giving a survey response rate of 75.9%. The study methodology is described in detail in an earlier article [19].

### Assessments

The diagnoses of mental disorders were established using the Composite International Diagnostic Interview version 3.0 (CIDI-3.0) [20]. We used the Computer Assisted Personal Interviewing (CAPI) version of the CIDI 3.0 in English and Chinese, and the paper and pencil interview (PAPI) version of the Malay CIDI. Diagnostic modules for lifetime and 12-month prevalence of mood disorders (MDD and Dysthymia), anxiety disorders (generalized anxiety disorder (GAD), and obsessive compulsive disorder (OCD)) and alcohol use disorders (alcohol abuse and alcohol dependence) were included in the survey. Diagnostic hierarchy rules and organic exclusion criteria were applied to all diagnoses. Help-seeking was assessed by analyzing the services module; help-seekers for the purposes of this study were defined as those who had consulted with any formal healthcare provider (i.e. psychiatrist, GP or any other medical doctor as only these healthcare professionals in Singapore can prescribe psychotropic drugs). The pharmacoepidemiology module of CIDI-3.0 was used to establish the use of ADs and BZDs in the population. The key questions, “Did you take any type of prescription medicine in the past 12 months for problems with your emotions, substance use, energy, concentration, sleep, or ability to cope with stress? Include medicines even if you took them only once” was asked to all respondents regardless of diagnostic status. A respondent booklet with names of various psychotropic medications was used to aid recall. Interviewers were also trained to encourage respondents to refer to prescriptions/medications to aid recall and provide precise answers. The raw data was reviewed, cleaned and medications were classified into different groups by a research assistant working closely with the principal investigator and co-author (SAC), who is an experienced research psychiatrist in Singapore.

We used a modified version of the CIDI 3.0 checklist of chronic medical conditions to capture information on chronic physical disorders which were considered prevalent in Singapore’s population. Socio-demographic information was collected using a structured questionnaire.

Disability was assessed with the Sheehan Disability Scale (SDS) which examined functioning in work, household, relationship, and social roles in the worst month of the past year. The Quick Inventory of Depressive Symptomatology Self-Report (QIDS-SR) [21], included in the CIDI 3.0 assessed symptom severity in MDD during the worst month of the previous year. Transformation rules developed for the QIDS-SR were used to convert the scores into clinical severity scores and categories of the Hamilton Rating Scale of Depression (HAMD) [22,23].

#### Statistical analyses

All estimates were weighted to adjust for over sampling and post-stratified for age and ethnicity distributions between the survey sample and the Singapore resident population in 2007. Estimates of the prevalence of ADs and/or BZDs usage were expressed in weighted percentages with standard error (SE/100). Chi square (*χ*^2^) tests were used to compare the prevalence rates between the groups. A series of multiple logistic regression models were used to generate odds ratios (ORs) and 95% confidence intervals using consumption of ADs, BZDs and ADs and/or BZDs as the main outcome variables and age group, ethnicity, gender, marital status, education, employment, help seeking, presence of chronic physical disorder, alcohol use disorder, anxiety disorders, bipolar disorder, dysthymia and MDD as the predictor variables. Statistical significance was evaluated at the p < 0.05 level using two-sided tests. All statistical analyses were carried out using the Statistical Analysis Software (SAS) System version 9.2 (Cary, NC).

## Results

The mean age of respondents was 43.9 years and the sample consisted of almost equal proportions of males and females. Table 1 shows the sociodemographic distribution of the respondents.

**Table 1:** Socio-demographic characteristics of the study sample (N = 6616)

	**Unweighted**		**Weighted**	**Population figures by Singapore census, 2010**
	**n**	**%**	**% (SE)**	**%**
Age				
Mean (SE), SD	42.0	14.5	43.9(0.3)	
Age Group				
18-34	2293	34.7	31.7(0.0)	31.2
35-49	2369	35.8	34.1(0.0)	32.2
50-64	1542	23.3	23.1(0.0)	25.1
65+	412	6.2	11.1(0.0)	11.4
Ethnicity				
Chinese	2006	30.3	76.9(0.0)	74.1
Malay	2373	35.9	12.3(0.0)	13.4
Indian	1969	29.8	8.3(0.0)	9.2
Others	268	4.1	2.4(0.0)	3.3
Gender				
Female	3317	50.1	51.5(0.9)	49.3
Male	3299	49.9	48.5(0.9)	50.7
Marital Status				
Never Married	1825	27.6	28.9(0.6)	
Currently Married	4290	64.9	62.4(0.8)	
Divorced/Separated	262	4.0	4.2(0.4)	
Widowed	237	3.6	4.4(0.4)	
Education				
Pre-primary/Primary	1236	18.7	20.2(0.7)	
Secondary	1975	29.9	27.6(0.8)	
Pre-u/Junior College/Diploma	1342	20.3	22.4(0.7)	
Vocational	721	10.9	7.9(0.4)	
University	1342	20.3	21.9(0.7)	
Employment				
Employed	4594	71.5	71.0(0.8)	
Economically inactive*	1522	23.7	24.5(0.7)	
Unemployed	313	4.9	4.5(0.4)	
Personal annual income				
Below S$20000	3392	54.0	51.3(0.8)	
S$20000 - 49999	1924	30.7	31.2(0.8)	
S$50000 & above	962	15.3	17.5(0.7)	

*Economically Inactive refers to retirees, housewives and students.

### Antidepressants/benzodiazepines use

The overall prevalence estimates for ADs and BZDs use during the 12 months prior to the interview were 1.1% and 1.2% respectively. In all, 2.0% had used ADs and/or BZDs. More than one-quarter of the people using ADs (26.7%) also used BZDs. The prevalence rates of ADs and BZDs use were significantly different across ethnicity, education and employment status. ‘Help-seeking for emotional problems’ was strongly associated with the use of ADs and BZDs. The unadjusted prevalence estimates revealed that a diagnosis of any mental disorder (alcohol use disorder, anxiety disorder, bipolar disorder, dysthymia or MDD was associated with more prevalent use of ADs and BZDs (Table 2).

**Table 2:** 12-month prevalence of antidepressants and benzodiazepines in the population by sociodemographic and clinical factors

		**AD**					**BZD**					**AD and/or BZD#**			
		**(n = 57)**					**(n = 60)**					**(n = 104))**			
**Variables**	**N**	**%**	**(SE)**	***X***^**2 **^**(DF)**	**P value**	**N**	**%**	**(SE)**	***X***^**2 **^**(DF)**	**P value**	**N**	**%**	**(SE)**	***X***^**2 **^**(DF)**	**P value**
All respondents		1.09	0.2				1.15	0.2				1.95	0.26		
Age Group															
18-34	17	0.48	0.17	5.7(3)	0.13	26	1.02	0.28	1.8(3)	0.62	40	1.46	0.32	1.6(3)	0.66
35-49	20	1.07	0.32			21	1.51	0.39			37	2.22	0.46		
50-64	17	1.6	0.48			11	0.77	0.32			23	1.99	0.53		
65+	3	1.8	1.03			2	1.2	0.85			4	2.41	1.19		
Ethnicity															
Chinese	22	1.18	0.26	7.9(3)	0.054	24	1.23	0.25	17.5(3)	0.0006	40	2.07	0.33	23.5(3)	<.0001
Malay	11	0.45	0.14			12	0.5	0.14			19	0.79	0.18		
Indian	19	0.94	0.22			16	0.81	0.2			33	1.64	0.28		
Others	5	1.84	0.86			8	3.24	1.17			12	4.86	1.41		
Gender															
Male	23	0.97	0.27	0.3(1)	0.56	27	1.15	0.3	0(1)	1	44	1.73	0.36	0.7(1)	0.42
Female	34	1.2	0.3			33	1.15	0.26			60	2.15	0.38		
Marital Status															
Single	15	1.18	0.36	3.6(2)	0.16	18	1	0.31	0.4(2)	0.83	31	2.14	0.47	3.1(2)	0.21
Married	34	0.88	0.23			38	1.24	0.27			61	1.67	0.31		
Divorced/Separated/Widowed	8	2.28	1.08			4	1.01	0.6			12	3.29	1.23		
Education															
Pre-Primary/Primary	15	1.92	0.65	11.3(4)	0.02	5	0.44	0.27	4.5(4)	0.34	17	2.12	0.67	2.4(4)	0.66
Secondary	15	1.44	0.45			17	1.43	0.47			28	2.34	0.57		
Pre-U/JC/Diploma	12	0.57	0.25			16	1.33	0.42			26	1.7	0.46		
Vocational	4	0.25	0.13			6	0.74	0.45			9	0.93	0.46		
University	11	0.71	0.3			16	1.42	0.45			24	1.9	0.51		
Employment Status															
Employed	30	0.73	0.18	8.3(2)	0.02	41	0.99	0.21	1.4(2)	0.50	64	1.57	0.26	4.9(2)	0.09
Econ. Inactive*	18	1.82	0.59			13	1.52	0.53			26	2.71	0.7		
Unemployed	5	2.73	1.45			2	1.71	1.19			6	3.59	1.67		
Help Seeking															
No	21	0.41	0.13	192.9(1)	<.0001	19	0.36	0.12	266.6(1)	<.0001	39	0.76	0.18	346.4(1)	<.0001
Yes	36	12.94	2.7			41	14.88	2.82			65	22.57	3.27		
Chronic physical disorder															
No	20	0.8	0.23	2.7(1)	0.1	21	0.74	0.2	6.0(1)	0.01	36	1.32	0.28	7.8(1)	0.005
Yes	37	1.47	0.36			39	1.71	0.38			68	2.79	0.48		
*Mental disorders*															
Alcohol use disorder															
Never	50	1.06	0.2	1.2(2)	0.55	49	1	0.19	17.7(2)	0.0001	88	1.77	0.26	15.4(2)	0.0004
Lifetime,	5	1.93	1.33			8	4.56	2.13			11	6.15	2.47		
12-month	2	1.33	0.96			3	6.92	4.98			5	8.26	5.05		
Anxiety disorder															
Never	46	0.97	0.2	26.5(2)	<.0001	47	0.98	0.19	24.3(2)	<.0001	85	1.71	0.25	32.3(2)	<.0001
Lifetime,	9	6.22	2.84			7	6.4	2.96			12	10.2	3.63		
12-month	2	1.11	0.85			6	4.6	2.73			7	5.38	2.83		
Bipolar disorder															
Never	55	1.09	0.2	-	-	54	1.1	0.2	8.3(2)	0.02	96	1.9	0.26	4.3(2)	0.12
Lifetime,	0	-	-			1	6.18	5.95			1	6.18	5.95		
12-month	2	1.32	0.97			5	4	1.9			7	5.32	2.19		
Dysthymia															
Never	55	1.05	0.2	16.8(1)	<.0001	57	1.13	0.2	9.4(1)	0.002	100	1.89	0.26	19.8(1)	<.0001
12-month	2	15.68	12.51			3	7.45	4.94			4	21.27	12.84		
Major depressive disorder															
Never	38	0.8	0.18	49.6(2)	<.0001	42	0.85	0.18	46.6(2)	<.0001	74	1.48	0.24	67.1(2)	<.0001
Lifetime,	6	3.52	1.8			6	4.47	1.99			11	6.92	2.43		
12-month	13	9.17	3.3			12	8.54	3.21			19	13.47	3.88		
Any mental disorders															
Never	27	0.73	0.19	29.9(2)	<.0001	27	0.67	0.17	50.6(2)	<.0001	51	1.24	0.24	64.5(2)	<.0001
Lifetime,	14	2.94	1.08			13	3.89	1.26			23	6.13	1.56		
12-month	16	4.96	1.73			20	6.1	1.89			30	8.9	2.23		

^#^Those who were prescribed either Antidepressants OR Benzodiazepine or were prescribed both Antidepressants AND Benzodiazepine.

Lifetime prevalence does not include 12-month prevalence.

*Economically Inactive refers to retirees, housewives and students.

Help-seeking significantly increased the use of ADs and BZDs in subjects with a lifetime or 12-month diagnosis of any mental disorder. For help-seekers with alcohol use disorder, the use of BZDs was much higher than the use of ADs. About one third (33.2%) of the help-seekers with lifetime alcohol use disorder used BZDs and about half (45.3%) of those with 12-month alcohol use disorder used BZDs. For help-seekers with anxiety disorder, the use of ADs was comparable to the use of BZDs in respondents with lifetime prevalence whereas the use of BZDs was much higher than the use of ADs among those with 12-month anxiety disorder. For help-seekers with bipolar disorder the use of BZD was much higher than the use of ADs among respondents with both life-time and 12-month disorder. For help-seekers with MDD, the use of ADs was comparable to the use of BZDs in respondents with both lifetime and 12-month disorder (Table 3).

**Table 3:** Individuals being prescribed antidepressants or benzodiazepines during the last 12 months by diagnostic category and help-seeking category

		**No help-seeking**		**Help-seeking**	
**Mental disorder**	**Prevalence**	**AD**	**BZD**	**AD**	**BZD**
		**%**	**%**	**%**	**%**
Alcohol use disorder	Never	0.42	0.33	12.71	13.22
	Lifetime	0.16	1.49	18.47	33.18
	12-month	0.00	0.61	9.43	45.30
Anxiety disorder	Never	0.37	0.32	12.80	13.95
	Lifetime	2.12	2.08	24.98	26.17
	12-month	0.45	0.83	2.84	14.62
Bipolar disorder	Never	0.41	0.36	14.03	15.16
	Lifetime	0.00	0.00	0.00	12.00
	12-month	0.00	1.03	4.93	12.13
Dysthymia	Never	0.37	0.36	13.18	14.96
	12-month	-(*)	2.95	3.71	11.93
Major depressive disorder	Never	0.33	0.29	11.34	13.36
	Lifetime	1.50	2.56	10.57	11.15
	12-month	2.65	0.55	26.34	29.55
No Disorder		0.30	0.26	12.69	11.98

Antidepressants — %/12 months (SE ranging from 0.13 to 12.3).

Benzodiazepines — %/12 months (SE ranging from 0.12 to 25.0).

Lifetime prevalence does not include 12-month prevalence.

*not reported due to the small number of people with dysthymia not seeking help.

There were significant differences in the use of ADs (*χ*^2^ = 36.7, p value < 0.0001) based on the type of professionals that the respondents had consulted for their ‘emotional or mental health problems’ in the 12 months preceding the interview (Table 4). Chi-square test revealed that the ratings of the Hamilton Depression Rating Scale (HAM-D) and Sheehan disability scale (SDS) were not associated with the use of any of the medications.

**Table 4:** Percentage of subjects taking antidepressants or benzodiazepines, within each source of help (Psychiatrist, General practitioner, Other medical doctor)

	**Psychiatrist**		**General practitioner**		**Other medical doctor**		
	**N**	**%**	**N**	**%**	**N**	**%**	**P value**
Antidepressant (%)	32	21.39	5	2.37	2	4.15	<.0001
Benzodiazepine (%)	27	17.13	18	15.99	2	4.24	0.14
Antidepressants or Benzodiazepines (%)	50	30.06	21	17.95	3	6.32	0.003

### Multivariate analysis

Table 5 shows the results of the multivariate logistic analysis. ‘Help-seeking for emotional or mental health problems’ was the most important predictor for the use of ADs and BZDs —help-seekers were much more likely to use ADs and BZDs than non-help-seekers in the previous 12 months. Ethnicity was associated with the use of BZDs; Malays’ usage of BZDs was significantly lower than that of the Chinese. Those who were married were less likely to use ADs compared to those who were single. Respondents who had pre-primary or primary education were 3.5 times more likely to use ADs than respondents with university education. Age group, gender and employment status were not associated with the use of either ADs or BZDs.

**Table 5:** Logistic regression analysis on sociodemographic and clinical factors associated with the prescription of antidepressants/benzodiazepines/both

	**AD**			**BZD**			**AD and/or BZD**		
**Variable**	**OR**	**(95% CI)**	**p**	**OR**	**(95% CI)**	**p**	**OR**	**(95% CI)**	**p**
Age Group									
18-34	Ref.			Ref.			Ref.		
35-49	2.91	(0.75,11.35)	0.12	1.27	(0.42,3.85)	0.67	1.91	(0.75,4.82)	0.17
50-64	3.49	(0.82,14.76)	0.09	0.6	(0.14,2.59)	0.49	1.35	(0.44,4.09)	0.60
65+	4.67	(0.54,40.56)	0.16	1.4	(0.19,10.53)	0.75	2.2	(0.44,10.93)	0.34
Ethnicity									
Chinese	Ref.			Ref.			Ref.		
Malay	0.39	(0.15,1.01)	0.05	0.38	(0.16,0.88)	0.02	0.33	(0.16,0.67)	0.002
Indian	0.96	(0.46,2)	0.91	0.5	(0.23,1.06)	0.07	0.72	(0.41,1.28)	0.26
Others	1.15	(0.27,4.91)	0.85	0.81	(0.24,2.7)	0.73	1.14	(0.42,3.1)	0.79
Gender									
Male	Ref.			Ref.			Ref.		
Female	1.27	(0.48,3.36)	0.64	0.96	(0.42,2.18)	0.912	1.31	(0.66,2.62)	0.45
Marital Status									
Single	Ref.			Ref.			Ref.		
Married	0.29	(0.1,0.85)	0.02	1.13	(0.39,3.29)	0.83	0.43	(0.19,1)	0.05
Divorced/Separated/Widowed	0.42	(0.06,2.73)	0.36	0.69	(0.08,5.88)	0.73	0.58	(0.14,2.44)	0.46
Education									
Pre-Pri/Primary	3.53	(1.06,11.75)	0.04	0.52	(0.08,3.29)	0.48	1.73	(0.62,4.78)	0.29
Secondary	1.96	(0.57,6.68)	0.28	1.23	(0.41,3.67)	0.71	1.42	(0.59,3.43)	0.44
Pre-U/JC/Diploma	0.7	(0.16,3.03)	0.64	1.1	(0.37,3.28)	0.86	0.88	(0.35,2.22)	0.79
Vocational	0.29	(0.05,1.73)	0.17	0.57	(0.17,1.97)	0.38	0.43	(0.14,1.32)	0.14
University	Ref.			Ref.			Ref.		
Employment Status									
Employed	Ref.			Ref.			Ref.		
Econ. Inactive*	1.53	(0.67,3.53)	0.32	1.28	(0.57,2.89)	0.55	1.22	(0.63,2.36)	0.56
Unemployed	0.66	(0.13,3.31)	0.61	0.76	(0.15,3.76)	0.73	0.67	(0.22,2.03)	0.47
Help Seeking									
No	Ref.			Ref.			Ref.		
Yes	31.62	(13.36,74.83)	<.0001	34.38	(12.97,91.16)	<.0001	32.18	(15.87,65.26)	<.0001
Chronic Physical Disorder									
No	Ref.			Ref.			Ref.		
Yes	0.92	(0.36,2.39)	0.87	1.63	(0.69,3.85)	0.27	1.31	(0.65,2.65)	0.46
*Mental disorders*									
Alcohol use disorder									
Never	Ref.			Ref.			Ref.		
Lifetime	2.02	(0.6,6.72)	0.25	5.18	(1.77,15.16)	0.003	4.28	(1.7,10.78)	0.002
12-month	0.78	(0.07,8.89)	0.84	3.41	(0.55,21.06)	0.19	2.82	(0.57,14.04)	0.21
Anxiety disorder									
Never	Ref.			Ref.			Ref.		
Lifetime	4.05	(0.96,17.12)	0.06	3.48	(1.03,11.76)	0.04	4.49	(1.47,13.67)	0.008
12-month	0.13	(0.01,1.32)	0.08	0.88	(0.17,4.47)	0.87	0.62	(0.14,2.78)	0.53
Bipolar disorder									
Never	Ref.			Ref.			Ref.		
Lifetime	-		-	0.52	(0.08,3.47)	0.50	0.27	(0.04,1.86)	0.18
12-month	-		-	2.04	(0.45,9.28)	0.36	1.77	(0.51,6.17)	0.37
Dysthymia									
Never	Ref.			Ref.			Ref.		
12-month	0.98	(0.02,43.52)	0.99	0.45	(0.05,4.24)	0.48	0.91	(0.05,16.04)	0.95
Major depressive disorder									
Never	Ref.			Ref.			Ref.		
Lifetime	1.3	(0.23,7.17)	0.77	1.59	(0.36,7.08)	0.54	1.42	(0.4,5.05)	0.59
12-month	7.6	(2.28,25.39)	0.001	4.45	(1.5,13.19)	0.007	4.12	(1.63,10.45)	0.002

*Economically Inactive refers to retirees, housewives and students.

Lifetime prevalence does not include 12-month prevalence.

Respondents with a diagnosis of lifetime alcohol use disorder or lifetime anxiety disorder were more likely to use BZDs than those without these disorders. A diagnosis of 12-month MDD was associated with the use of ADs and BZDs. The multivariate analysis revealed that the presence of a bipolar disorder, dysthymia or chronic physical disorders did not have a predictive utility for the use of either ADs or BZDs.

## Discussion

The overall prevalence estimates for ADs and BZDs use during the 12 months prior to the interview were 1.1% and 1.2% respectively, with 2.0% using ADs and/or BZDs. These rates were lower than those found in the ESEMeD project where 4.38% of the respondents had taken ADs and 9.17% had used BZD in the past 12 months, while overall, 11.68% of their sample had used ADs or BZD [4]. Our rates were slightly lower than that of 2.0% AD use reported from the Israel National Health survey [8]. We would like to highlight that the rates of MDD as established in the SMHS (lifetime and 12-month prevalence estimates for MDD were 5.8% and 2.2%, respectively) [24,25] were lower than those reported by the ESEMeD project (lifetime and 12-month prevalence estimates for MDD were 12.8% and 3.9%, respectively) [26].

Sociodemographic factors that were associated with the use of ADs and BZDs included ethnicity, marital status, and education. Indicators of social exclusion such as not being married and low education have been associated with low social support and increased psychological distress and this may be correlated to higher utilization of ADs in these groups [27,28]. We did not find age or gender to be correlated with the use of ADs and BZDs.

Those seeking help from psychiatrists were significantly more likely to use ADs as compared to those seeking help from GPs or other medical doctors, while the use of BZDs was not significantly associated with their source of help-seeking. The US National Comorbidity Survey similarly found that individuals with a mood or anxiety disorder were more likely to receive ADs and anxiolytics from psychiatrists than from primary care physicians [12]. A recent questionnaire based study among psychiatrists and GPs in Belgium found that more psychiatrists than GPs are high prescribers of ADs (prescribed antidepressants to > 50% of their patients with depression) [29]. The authors suggested that this could be due to differences in the severity of depression among those seeking help from psychiatrists and GPs. However, our study did not find any association with either functional or symptom severity and use of ADs.

As with other studies [4,15], we found that a large proportion of those with 12-month MDD were not prescribed ADs. This could be due to a failure to recognize their distress as a mental health disorder, ignorance of available treatments or due to the considerable stigma associated with mental illnesses. Therefore, until people conceive that they require some help, they may not seek treatment. Even in those seeking help, various factors associated with the care-providers may further limit the prescription of ADs [30].

The adjusted analyses showed that the strongest predictor for the use of ADs and BZDs was “help-seeking” behavior. It is possible that those who are help-seeking had a sub-clinical presentation which while not meeting any DSM-IV diagnosis criteria, caused the respondents significant distress and functional impairment leading to the prescription. It is also possible that the medications were prescribed for other disorders that were not included in our study. Lastly, the question on help-seeking specifies, “seeking help for problems with your emotions, nerves, mental health or your use of alcohol or drugs”; thus it is possible that some respondents with a mental disorder failed to perceive their problems as such and sought help for other complaints such as somatic symptoms, and therefore ascribed a more physical cause to their illness. The use of ADs was more prevalent among respondents with 12-month prevalence of MDD than in respondents with lifetime prevalence of MDD. The Singapore Clinical Practice Guidelines for Depression [31] recommends that patients with a first episode of depression without psychotic symptoms should be treated with ADs at full treatment dose for 6–9 months after remission of symptoms and that patients who have a second episode of depression should be maintained on treatment for 1–2 years, this provides a possible explanation as to why only about 13% of those with lifetime MDD were using ADs. Lifetime or 12-month bipolar disorder was not associated with the use of ADs or BZDs. In a previous report, we found that the proportion of respondents prescribed treatment with antipsychotic, AD and/or mood-stabilizer among those with 12-month bipolar disorder were 1.6%, 1.3% and 7.1% respectively [32], reflecting a preference of prescribing mood stabilizer as the mainstay of psychopharmacotherapy for bipolar disorder Help-seeking respondents with both 12-month MDD and anxiety disorders were more likely to use BZDs than ADs. Studies have suggested that patients often consult physicians for somatic or sleep complaints and only a high degree of suspicion and careful screening would reveal the underlying mental disorder [33], thus symptomatic treatment would result in misdiagnosis and inadequate treatment of the primary disorder, which could result in adverse outcomes. Other studies have similarly found that BZDs are commonly prescribed to those with mood and anxiety disorders [4,34]. Most recent international treatment guidelines [35,36] further, recommend limiting the use of BDZs only in patients with primary major depression to those with pronounced anxiety or persistent insomnia that are not adequately relieved by ADs. International guidelines for anxiety disorders [35,37] currently recommend selective serotonin reuptake inhibitors (SSRIs) and serotonin/norepinephrine reuptake inhibitors (SNRIs) as the drugs of first choice, with BZDs as second-line option.

We found the use of BZDs to be significantly higher among those with lifetime alcohol use disorders. Alcohol dependence and the associated use of legal psychotropic drugs have been identified in several clinical studies [38-40]. Those with alcohol use disorders may use BZDs with alcohol or as a substitute when alcohol is unavailable. They may also self-medicate with BZDs to ease alcohol’s withdrawal symptoms or for comorbid anxiety disorders. A recent Treatment Episode Data Set (TEDS) report from US suggested that ‘since BZD abuse is most often a secondary substance of abuse, prescribing physicians may wish to screen for alcohol and other drugs of abuse and monitor patients more closely’ [41].

The limitations of our study include the fact that the data were entirely based on self-report and the willingness of the respondents to disclose the use of medications, thus our rates are possibly underestimates. Studies have demonstrated that there is good concordance between self-reported psychotropic medication use and information obtained from official prescription databases for most psychotropic drugs [42]. Our study did not establish non-pharmacological treatments, thus it may be possible that those with anxiety or mood disorder were receiving behavioral interventions which may have resulted in some of our findings. As this was a household study, we excluded those who were in institutions such as hospitals/nursing homes and prisons during the field period of the study and these populations may have a higher use of psychotropic medications.

The strengths of our study are the use of a structured standardized instrument and well trained interviewers who captured the information systematically and accurately. We also achieved a relatively high response rate of 75.9% which makes the findings generalizable to the resident population.

## Conclusions

In conclusion, we found that use of ADs and BZDs in our population was relatively low and that ‘help-seeking’ was the most important predictor of the use of ADs and BZDs. What needs to be further investigated is the appropriateness of the use of these psychotropic medications – a question which was beyond the scope of this study. However, it would seem there might be an underutilization of ADs among those with 12-month MDD (although we could not establish whether these respondents have received any form of psychotherapy), as well as a relatively high rate of BZDs use among those with alcohol use disorders. However, these findings need to be further researched in this population.

## Competing interests

The authors report no other financial or other relationship relevant to the subject of this article.

## Authors’ contributions

MS wrote the first draft of the article and was involved in study design and protocol writing. VYFH conducted the statistical analyses. JAV helped in study design, protocol writing and gave inputs into the article. EA helped in study design, protocol writing and gave inputs into the article. SA helped in study design, protocol writing and gave inputs into the article. All authors read and approved the final manuscript.

## Pre-publication history

The pre-publication history for this paper can be accessed here:

http://www.biomedcentral.com/1471-244X/13/231/prepub
